# Improved fetal blood oxygenation and placental estimated measurements of diffusion‐weighted MRI using data‐driven Bayesian modeling

**DOI:** 10.1002/mrm.28075

**Published:** 2019-11-19

**Authors:** Dimitra Flouri, David Owen, Rosalind Aughwane, Nada Mufti, Kasia Maksym, Magdalena Sokolska, Giles Kendall, Alan Bainbridge, David Atkinson, Tom Vercauteren, Sebastien Ourselin, Anna L. David, Andrew Melbourne

**Affiliations:** ^1^ School of Biomedical Engineering and Imaging Sciences King’s College London London United Kingdom; ^2^ Department of Medical Physics and Biomedical Engineering University College London London United Kingdom; ^3^ Institute for Women’s Health University College Hospital London United Kingdom; ^4^ Medical Physics University College Hospital London United Kingdom; ^5^ Centre for Medical Imaging University College London London United Kingdom; ^6^ University Hospital KU Leuven Leuven Belgium; ^7^ NIHR Biomedical Research Centre University College London Hospitals London United Kingdom

**Keywords:** Bayesian estimation, DECIDE, diffusion‐weighted MRI, registration, shrinkage prior

## Abstract

**Purpose:**

Motion correction in placental DW‐MRI is challenging due to maternal breathing motion, maternal movements, and rapid intensity changes. Parameter estimates are usually obtained using least‐squares methods for voxel‐wise fitting; however, they typically give noisy estimates due to low signal‐to‐noise ratio. We introduce a model‐driven registration (MDR) technique which incorporates a placenta‐specific signal model into the registration process, and we present a Bayesian approach for Diffusion‐rElaxation Combined Imaging for Detailed placental Evaluation model to obtain individual and population trends in estimated parameters.

**Methods:**

MDR exploits the fact that a placenta signal model is available and thus we incorporate it into the registration to generate a series of target images. The proposed registration method is compared to a pre‐existing method used for DCE‐MRI data making use of principal components analysis. The Bayesian shrinkage prior (BSP) method has no user‐defined parameters and therefore measures of parameter variation in a region of interest are determined by the data alone. The MDR method and the Bayesian approach were evaluated on 10 control 4D DW‐MRI singleton placental data.

**Results:**

MDR method improves the alignment of placenta data compared to the pre‐existing method. It also shows a further reduction of the residual error between the data and the fit. BSP approach showed higher precision leading to more clearly apparent spatial features in the parameter maps. Placental fetal oxygen saturation (FO_2_) showed a negative linear correlation with gestational age.

**Conclusions:**

The proposed pipeline provides a robust framework for registering DW‐MRI data and analyzing longitudinal changes of placental function.

## 1 INTRODUCTION

Quantitative diffusion‐weighted magnetic resonance imaging (DW‐MRI) parameters have been increasingly used to characterize abnormal placental microstructure.^1^, ^2^, ^3^, ^4^ Monitoring placental function using MRI may improve the understanding and diagnosis of placental insufficiency, which is a significant cause of perinatal morbidity and loss. DW‐MRI is becoming a powerful tool to obtain placenta perfusion‐related measures without the administration of a contrast agent.^5^, ^6^ DW‐MRI in combination with the intra‐voxel incoherent motion (IVIM) model provides a non‐invasive technique to assess tissue properties related to perfusion and flow. Another method for assessing placental function is $T_{2}$ relaxometry which provides information on the static tissue composition and intrinsic tissue $T_{2}$ value.^7^, ^8^ A recent study has proposed a joint placental model and acquisition, named Diffusion‐rElaxation Combined Imaging for Detailed placental Evaluation (DECIDE).^6^ The DECIDE model is a three‐compartment model of placental perfusion that combines $T_{2}$ relaxometry and DW imaging.

Placenta diffusion and relaxation imaging are quite susceptible to low signal‐to‐noise ratio (SNR) and motion artifacts due to maternal breathing and fetal movements.^9^, ^10^ Such movements can cause errors in the analysis of the data and image registration is thus required. Image registration of DW‐MRI data is very challenging due to image contrast variation dependent on the choice of echo time and diffusion weighting between the images.^11^ As a result, co‐registration of all functional images to a single target, eg, *b* = 0 ${s.\mathrm{mm}}^{-2}$ image, may not be very accurate especially for high b‐value images where the SNR is very low and signal is significantly attenuated.

Several image registration strategies have been developed to overcome the effect of motion and provide well‐aligned features across the images. Data‐driven registration methods based on principal component analysis (PCA) have been proposed for registering dynamic contrast‐enhanced MRI.^12^, ^13^ These methods rely on heuristic assumptions such that the contrast changes appear in more significant components and motion effects in the less significant components. This does not always bear true for all types of motion and structures in the image.

Another data‐driven PCA‐based groupwise method which registers quantitative MRI data has recently been proposed.^14^ However, this method is only applicable to data from a simplified mono‐exponential signal decay rather than data with an underlying complex signal decay such as placenta data acquired with DECIDE acquisition.^15^ Other reported registration methods that use a physical model to drive the registration process have also been proposed by other research groups.^15^, ^16^, ^17^, ^18^, ^19^, ^20^ Model‐driven methods eliminate the requirement to choose a target image and are robust to the intensity changes in the images. Furthermore, these approaches depend only on the underlying tissue physiology.

Many different approaches have been developed over the years to determine IVIM coefficients including least‐squares methods, optimal sampling, and Bayesian fitting.^21^, ^22^, ^23^, ^24^ Previous research on simulated data and on liver DW‐MRI has shown that least‐squares methods give noisy estimates especially for pseudo‐diffusion parameters, and fitting is ordinarily independent of spatial position which limits its applicability for assessing spatial features and heterogeneity.^24^ A Bayesian approach may reduce estimation uncertainty so that spatial features in parameter maps are more clearly apparent. The quantitative DECIDE‐based analysis of placental DW‐MRI allows the separate scrutiny of placental diffusion and perfusion information, without the need for contrast agents which are currently contraindicated in pregnancy.^6^, ^25^ A Bayesian‐based fitting method has not yet been proposed for the DECIDE model, which is the only current tissue‐specific model for placental imaging.

Assessing normal placental perfusion with gestational age is key to better understand differences linked to placental insufficiency. A recent study has shown changes in IVIM placental parameters, including perfusion and diffusion with gestational age.^4^ However, the variability in the measured parameters with gestational age has not previously been investigated for the DECIDE model.

Our primary contribution consists of a new framework which provides functional information of the placenta and study correlations between the DECIDE estimated placenta parameters and fetal growth. The aim of this paper is twofold. First, we present an iterative model‐driven registration (MDR) strategy which incorporates a placenta signal model to account for changes in image contrast. We then compare the MDR to a pre‐existing registration method used for dynamic contrast‐enhanced MRI data making use of PCA named progressive principal component registration (PPCR).^12^ Second, we extend the Bayesian shrinkage prior (BSP) approach originally proposed for the two‐compartment IVIM model^24^ to fit the advanced three‐compartment model DECIDE.

## 2 METHODS

### 2.1 Data

The study involved a cohort of 10 healthy women with a singleton pregnancy with no known placenta complications. Obstetric ultrasound scan confirmed normal fetal weight greater than the 10th centile and normal umbilical artery Doppler assessment done within 1 week of the MRI scan. The gestational ages ranged between 25+1 to 34+0 weeks+days with mean of 29+1 and standard deviation (SD) of 2+2. The study was approved by a local research ethics committee and written informed consent was obtained from each subject (London‐Hampstead Research Ethics Committee, REC reference 15/LO/1488).

### 2.2 MR imaging

MRI was performed on a 1.5T Siemens Avanto scanner (Siemens, Erlangen, Germany), in combinations from seven b‐values (**b**; 0, 50, 100, 150, 200, 400, 600 ${s.\mathrm{mm}}^{-2}$) and ten echo times ($T_{E}$) ($T_{E}$; 81, 90, 96, 120, 150, 180, 210, 240, 270, 300 ms). All $T_{E}$ were acquired at b = 0 ${s.\mathrm{mm}}^{-2}$ to allow $T_{2}$ fitting and all b‐values at $T_{E}$ = 96 ms. In addition, data were acquired at b‐value 50 ${s.\mathrm{mm}}^{-2}$ and 200 ${s.\mathrm{mm}}^{-2}$ for $T_{E}$ = (81, 96, 120, 150, 180, 210, 240 ms), to allow simultaneous sampling of diffusivity and relaxivity. Subjects were imaged using a pulsed gradient spin‐echo with an EPI readout. The dynamic series consisted of 41 volumes per subject. Other parameter settings were as follows: repetition time = 3900 ms, field of view $=\;402\;\times \;479\;\times \;437\;{\text{mm}}^{3}$, reconstructed matrix 156×192×26 and temporal resolution 4.1 s. The total acquisition time was approximately 20 min. Subjects were advised to breathe normally throughout the DW acquisition.

### 2.3 Model‐driven registration

**Figure 1:**
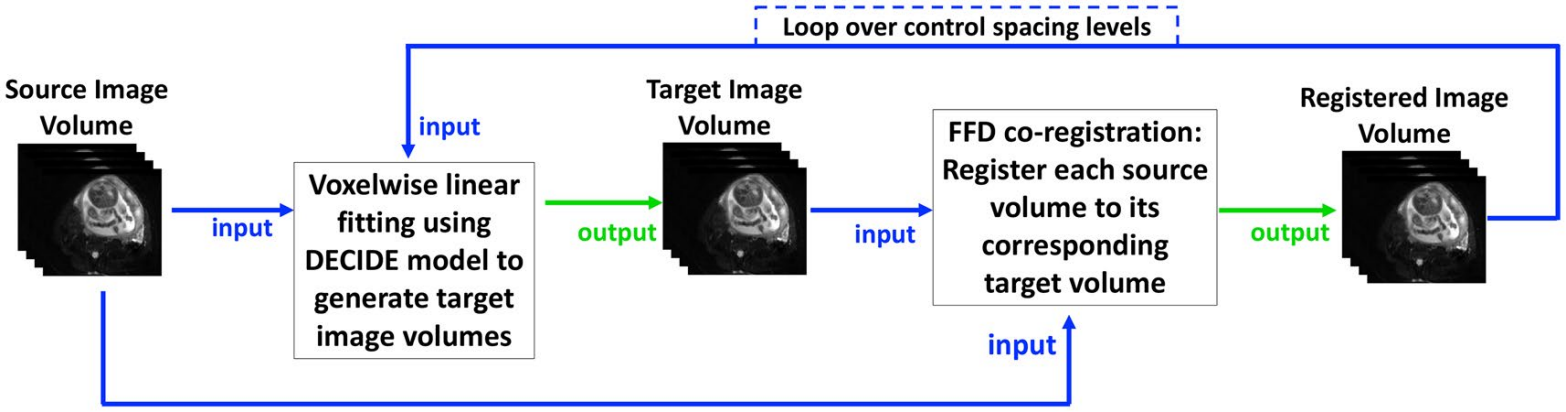
Diagram illustrating the process of the MDR method

The basic framework of the MDR method contains three steps which are described in detail below: (a) Fit the DECIDE model with linear inversion to the measurement volumes; (b) Synthesize target data for each measurement volume from the fitted model; (c) Register each measurement volume to the corresponding synthetic target volume. This process is summarized in Figure 1.

#### 2.3.1 Generation of target image volumes

##### DECIDE signal model

The DECIDE signal model^6^ is of the form:

(1) $$S\left(b,\;T_{E}\right)\;=\;S_{0}\left[fe^{-bd^{*}-T_{E}r_{2}^{f_{b}}}+\;\left(1-f\right)e^{-bd}\left(ve^{-T_{E}r_{2}^{m_{b}}}+\;\left(1-v\right)e^{-T_{E}r_{2}^{t}}\right)\right],$$

where S is the measured MRI signal and $S_{0}$ is the signal with no diffusion weighting (ie, *b* = 0). The five independent model parameters are the rapid‐perfusing volume fraction f, diffusivity d, pseudo‐diffusivity $d^{*}$, placental fetal blood relaxation $r_{2}^{f_{b}}\;=\;1/T_{2}^{f_{b}}$, and slow‐perfusing blood volume fraction v. As in Melbourne et al,^6^ we used literature‐based values for maternal blood relaxation $r_{2}^{m_{b}}$ and tissue relaxation $r_{2}^{t}$ of ${\left(240\;\text{ms}\right)}^{-1}$ and ${\left(46\;\text{ms}\right)}^{-1}$ respectively.^26^, ^27^, ^28^

##### DECIDE model fitting

Non‐linear least‐squares methods are the most commonly used algorithms to fit models to MR data. The process is slow and it is therefore computationally inefficient for estimation of image‐wide parameter estimates.

An efficient method to estimate model parameters is the use of linear least‐squares (LLS) by solving a linear system of equations. The LLS method has been used for DCE‐MRI and positron emission tomography.^29^, ^30^ In this work, we adopted a similar approach to produce parameter estimates for the DECIDE model since it is computationally intensive to use the standard method of fitting the solution of Equation (1) in the MDR approach.

First, we redefined the parameters in Equation (1) as follows:

(2) $$S\left(b,\;T_{E}\right)\;=\;\lambda_{1}e^{-bd^{*}-T_{E}r_{2}^{f_{b}}}+\lambda_{2}e^{-bd-T_{E}r_{2}^{m_{b}}}+\lambda_{3}e^{-bd-T_{E}r_{2}^{t}},$$

where $\lambda_{1}\;=\;S_{0}f$, $\lambda_{2}\;=\;S_{0}\left(1-f\right)v$ and $\lambda_{3}\;=\;S_{0}\left(1-f\right)\left(1-v\right)$. Assuming the parameters $d^{*}$ and $r_{2}^{f_{b}}$ are known, Equation (2) is a multiple linear regression model. Literature values for $d^{*}$ and $r_{2}^{f_{b}}$ were set to $0.073\;{\text{mm}}^{2}s^{-1}$ and $144.89\;{\text{ms}}^{-1}$ respectively.^6^ If the data $S(b,\;T_{E})$ are measured at N different b‐values and echo times then Equation (2) leads to a system of N linear equations. They can be summarized as a matrix equation S=Ax where $S\;=\;[S\left(b_{0},\;T_{E_{0}}\right),\;\cdots ,\;\left(b_{N},\;T_{E_{N}}\right)]$ is an array holding the measured signals, $x\;=\;\left[\lambda_{1},\;\lambda_{2},\;\lambda_{3}\right]$ contains the unknowns and A is an N × 3 matrix with the exponential terms. The matrix equation can be solved using standard methods for LLS problems. We then derived the physiological parameters $S_{0},\;f$ and v from the given $\lambda_{1},\;\lambda_{2}$ and $\lambda_{3}$:

(3) $$v\;=\;\frac{\lambda_{2}}{\lambda_{2}+\lambda_{3}},\;f\;=\;\frac{\lambda_{1}}{\lambda_{1}+\lambda_{2}+\lambda_{3}},\;S_{0}\;=\;\frac{1}{\lambda_{1}+\lambda_{2}+\lambda_{3}}$$

By fitting Equation (2) voxelwise to the data, we thus build up a series of synthetic model volumes. These volumes typically preserve the structure and the expected signal variations due to **b**−value and $T_{E}$ modulation and are then used as target image volumes in the registration process.

#### 2.3.2 Registration algorithm

The model‐based formulation of the MDR method eliminates the requirement of choosing a single target image. Each original unregistered image volume in the quantitative imaging series is registered to its corresponding target image volume generated as described above. The pairwise co‐registration is performed using a highly optimized C++ implementation of free‐form deformation (FFD) registration.^31^

#### 2.3.3 Implementation details

The DECIDE linear model fitting procedure was implemented in MATLAB (The MathWorks, Natick, Massachusetts) and run on a laptop computer with 16 GB memory and 3.1 GHz Intel Core i5 processor.

A B‐spline transformation has been used where the control point grid was subjected to a multiresolution scheme. The model fitting and registration steps are alternated three times with the spacing between the FFD control points decreasing at each iteration (10×10×10,5×5×5,2.5×2.5×2.5 voxels).

#### 2.3.4 Evaluation of registration performance

##### Image processing

Quantitative assessment was carried out on regions of interest (ROI) of the placenta. The placenta ROIs were manually segmented (ITK‐SNAP Version 3.6.0, 2017) from the unregistered baseline image (lowest **T_E_**, no diffusion weighting). Voxel‐by‐voxel fitting was performed with a nonlinear DECIDE model fit using a Levenberg–Marquardt algorithm (The MathWorks, Natick, Massachusetts) applied to Equation (1). One should note that in order to linearise Equation (1), we assumed that $d^{*}$ and $r_{2}^{f_{b}}$ are known. However, for the quantitative analysis $d^{*}$ and $r_{2}^{f_{b}}$ parameters need to be estimated and therefore the standard nonlinear fitting has been used.

##### Clinical data

The performance of MDR is compared to PPCR algorithm as described in Melbourne et al.^12^ To facilitate a fair comparison between the two registration methods, the FFD registration in PPCR was used with the same tuning as described in Section 2.3.2. The registration quality in the clinical data was assessed qualitatively by visual comparison of parameter maps and images before and after motion correction. In addition to the qualitative assessment, registration quality was also assessed by computing the normalized root mean square error between the data and the fit.

##### Simulated motion

To evaluate how the MDR method performs in a setting with known ground truth, we chose a real dataset with minimal motion corruption to be used as a point of reference. This dataset was then deformed by applying a ground truth motion derived from a clinical dataset with significant movement corruption. The simulated motion yields an average placenta deformation of 2.11 mm in *x*‐direction, 2.54 mm in y‐direction and 1.56 mm in *z*‐direction.

For each reconstruction $p_{i}$ of a parameter $p\;=\;f,\;d,\;d*,\;T_{2}^{f_{b}}$, v, the relative error was determined as:

(4) $$E\left(p\right)\;=\;\frac{p_{i}-p}{p}$$

MDR quality was also assessed visually by comparing reconstructed parameter maps against the exact parameter maps.

### 2.4 Data‐driven Bayesian modelling

#### 2.4.1 Least‐squares based approach

Voxelwise least‐squares (LSQ) parameter estimates were obtained using a Levenberg–Marquadt algorithm (The MathWorks, Natick, Massachusetts) applied to Equation (1). The LSQ fitting routine initialized with parameter estimates from model‐fitting results obtained from average placental ROIs signal curves as done in Melbourne et al.^6^ To stabilize the fitting when computing voxelwise estimates, the following constraints were chosen: 0<f<1 (no units), $0\;<\;d\;<\;1\;({\mathrm{mm}}^{2}s^{-1})$, $0\;<\;d^{*}\;<\;1\;\left({\mathrm{mm}}^{2}s^{-1}\right)$, $0\;<\;T_{2}^{f_{b}}\;<\;500$ (ms), 0<v<1 (no units).

#### 2.4.2 Bayesian shrinkage prior

We extend^24^ to iteratively adapt our DECIDE voxelwise fits based upon a hierarchical prior distribution generated from the placenta ROI‐informed statistics. The ROIs contain voxels with similar values and therefore a spatial correlation is introduced in the prior distribution. If the signal from a voxel is dominated by noise, parameter estimation is more heavily weighted by the prior distribution; while if SNR is high then the data has more influence in the parameter estimation. The idea of BSP method as described in Orton et al^24^ is to maximize a joint posterior probability of DECIDE parameters, given the observed data:

(5) $$p\left(\theta_{1:M},\;\mu ,\;\Sigma_{\mu }|S_{1:M}\right)\propto \prod_{i\;=\;1}^{M}p\left(S_{i}|\theta_{i}\right)p\left(\theta_{i}|\mu ,\;\Sigma_{\mu }\right),$$

where $\theta_{i}=\left[f_{i}\;d_{i}\;d_{i}^{*}\;r_{2}^{f_{b}}\;v_{i}\right]$ for voxel i, * M* is the number of voxels, $\mu ={\left[\mu_{f}\;\mu_{d}\;\mu_{d^{*}}\;\mu_{r_{2}^{f_{b}}}\;\mu_{v}\right]}^{T}$ is the ROI mean, $\Sigma_{\mu }$ is a 5×5 covariance matrix of μ, and $S_{i}$ is the signal at the voxel i. Model parameters are modified to ensure their values fall in a sensible range. Specifically, the prior distribution is defined over the transformed parameter D = log(d) such that $d\;=\;e^{D}$ subject to d > 0 (similarly for $d^{*}$ and $r_{2}^{\mathrm{fb}}$), and for f; the prior distribution is defined over the transformed parameter F = log(f)−log(1−f) such that $f\;=\;e^{F}/\left(1+e^{F}\right)$ subject to 0 < f < 1 (similarly for v). The likelihood function $p(S_{i}|\theta_{i})$ is a multivariate conditional probability that takes the form^24^:

(6) $$p\left(S|f,\;d,\;d^{*},\;r_{2}^{f_{b}},\;v\right)\propto {\left[S^{T}S-{\left(S^{T}g\right)}^{2}/\left(g^{T}g\right)\right]}^{-N/2},$$

where g is the expected signal vector normalized by the baseline signal $S_{0}$, and N is the number of measurements in DECIDE acquisition.^6^ The shrinkage prior function $p(\theta_{i},\;\mu ,\;\Sigma_{\mu })$ subjects to a multivariate Gaussian distribution, the formulation of which is given by:

(7) $$\theta_{i}∼N\left(\mu ,\;\Sigma_{\mu }\right).$$

In order to perform inference with the shrinkage priors, we used Markov Chain Monte Carlo (MCMC) with Gibbs sampling as in,^24^, ^32^ allowing us to infer voxelwise parameter values, *θ*, and ROI hyperparameters shared among voxels, μ and $\Sigma_{\mu }$. MCMC was initialized with the voxelwise LSQ estimates, then updated separately as described in the Appendix.

#### 2.4.3 Evaluation of estimation methods

For each subject a placenta ROI was drawn as described in Section 2.3.4. DECIDE fitting was then performed using the LSQ method and with the BSP approach. As well as a visual inspection of the parameter maps, summary statistics such as the median, 25^th^ and 75^th^ interquartile range were computed.

In Orton et al^24^ has demonstrated that the image filtering of the source images did not lead to smoother LSQ estimates compared to BSP parameter maps. Based on this observation, a Gaussian filter with *SD* of 1.0 (voxel) was applied to our data to smooth the source images prior to the LSQ fitting.

The relationships between DECIDE functional parameters ($f,\;d,\;d^{*}$, ${\text{FO}}_{2}$, v) and GA were evaluated by means of regression analysis, with *y* = *b*GA + *a*, where *y* denotes the measured functional parameter. The correlations between DECIDE parameters and GA were assessed using Pearson’s correlation coefficient. Significance level was set at 5%.

## 3 RESULTS

### 3.1 Registration

**Figure 2:**
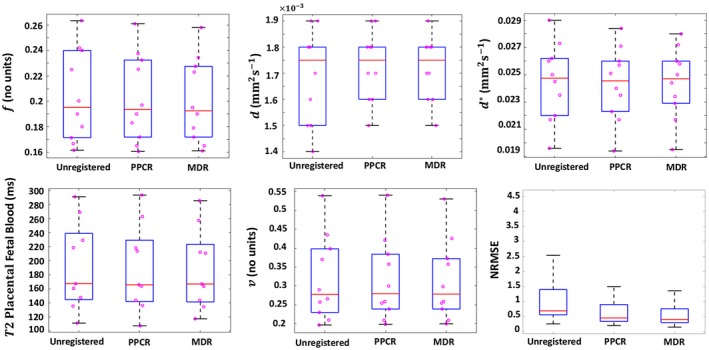
Box‐plots summarizing results for DECIDE parameters over 10 subjects. Each plot shows: the median (redline), the $25^{\mathrm{th}}$ and $75^{\mathrm{th}}$ percentile (blue box), individual means (pink circle) and the full data extent (black dashedline)

Figure 2 shows box‐plots for DECIDE parameter estimates over the 10 subjects included in this study. Results are presented for the median voxel value over the placenta ROIs and the mean estimates derived from the individual ROI of each subject. Analysis showed a reduction of error in registered data. The interquartile ranges were lower with MDR although median errors were similar for the registered and unregistered data.

Table 1 gives the mean and *SD* for DECIDE parameter estimates for the motion free data as well as before and after registration. The MDR method improves the accuracy and precisions of the parameter estimates.

**Table 1:** Comparison of DECIDE estimated parameters before and after MDR. Results are presented as mean value (SD)

Parameter	Unregistered data	Data with simulated motion	Registered data
f (no units)	0.225 (0.21)	0.251 (0.28)	0.218 (0.22)
$d\;({\text{mm}}^{2}s^{-1})$	0.0015 (0.0003)	0.0018 (0.0007)	0.0016 (0.0004)
$d^{*}\;\left({\text{mm}}^{2}s^{-1}\right)$	0.0385 (0.018)	0.0476 (0.29)	0.0368 (0.020)
$T_{2}$ Placental Fetal blood (ms)	181.1 (25.7)	142.1 (36.7)	170.4 (26.9)
v (no units)	0.306 (0.051)	0.369 (0.068)	0.325 (0.055)

Relative errors with respect to motion free data are presented in Table 2. Registration resulted in a decrease of error in all the parameters.

**Table 2:** Comparison of the relative error in the DECIDE parameters for the data with added simulated motion before and after MDR. Relative error was calculated with respect to original data

Parameter	Data with simulated motion	Registered data
f (no units)	0.12	0.03
$d\;({\text{mm}}^{2}s^{-1})$	0.20	0.07
$d^{*}\;\left({\text{mm}}^{2}s^{-1}\right)$	0.24	0.08
$T_{2}$ Placental Fetal blood (ms)	0.22	0.06
v (no units)	0.21	0.06

**Figure 3:**
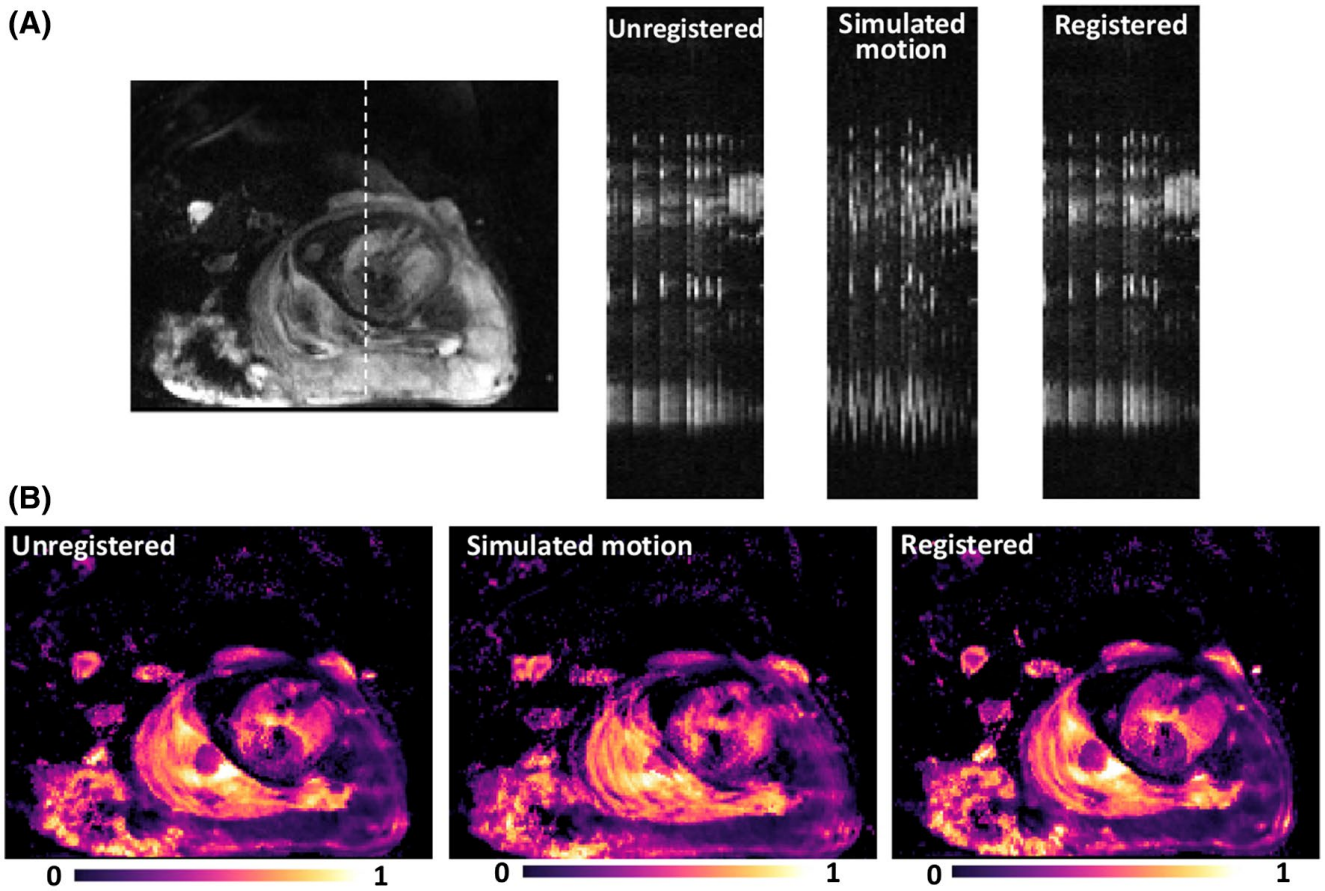
A, Profile of dynamic image stacks (cuts) of a single column of each image in the dynamic series. Coronal view for anatomical reference, a dashed line indicates the location of the cut in unregistered data (no registration), data with added simulated nonrigid motion and data registered with MDR. B, Fetal blood volume fraction maps for original data, data with added simulated nonrigid motion and data registered with MDR

Figure 3 show the effect of the MDR algorithm in original data and the data with added simulated nonrigid motion before and after motion correction with the MDR. Registration with MDR shows that misalignment due to motion were reduced and produces sharpened parameter maps (see the arrows shown on the relevant figure).

**Figure 4:**
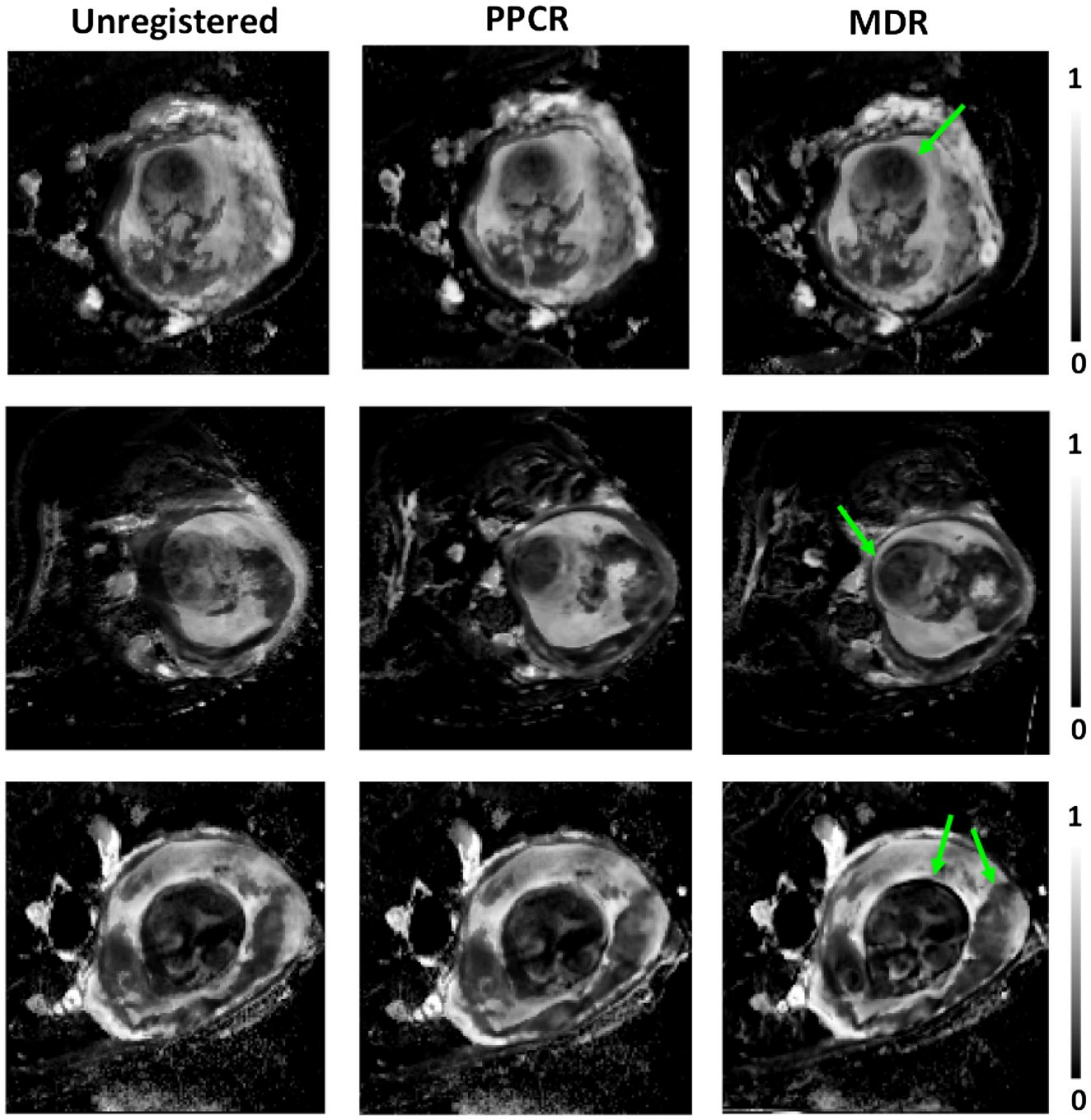
A comparison of fetal blood volume fraction maps in 3 subjects for unregistered, registered with PPRC and registered with MDR. The comparison shows further reduction of the motion artifacts and sharper delineation of organ boundaries (see arrows) on maps calculated with MDR

Figure 4 illustrates the effect of image registration before and after motion correction on fetal blood volume fraction maps. PPCR reduces the motion‐induced blurring that is visible on the uncorrected maps. However, the improvement was limited compared to MDR. Registration with MDR shows improvement of anatomical delineation and precision of parameter maps.

**Figure 5:**
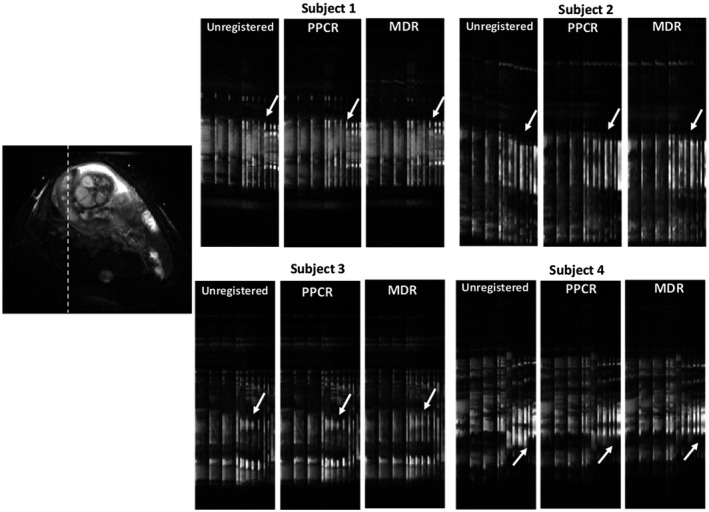
Effect of registration in superior‐inferior direction in 4 subjects. An axial view is presented for anatomical reference with a dashed line indicates an example of location of the cuts. Arrows indicate further alignment with MDR method where registration with PPCR showed some residuals

Figure 5 shows examples of registration in 4 subjects. Misalignments due to respiratory motion are visible when no registration is applied. However, it is observed that they are reduced after applying registration with PPCR and MDR. Arrows pointing up on the figure show that after applying registration with MDR images were almost perfectly aligned where in some cases registration with PPCR showed some residuals due to uncorrected motion.

The time taken by the linear LSQ using the linearized model in Equation (2) to find the optimum parameters and create target imaging volumes is about 250 times less than that required for a conventional nonlinear LSQ method. The linear LSQ technique reduced the calculation times for a 156×192×26 MR volume from 16 min to 4 s.

### 3.2 BSP fitting

**Figure 6:**
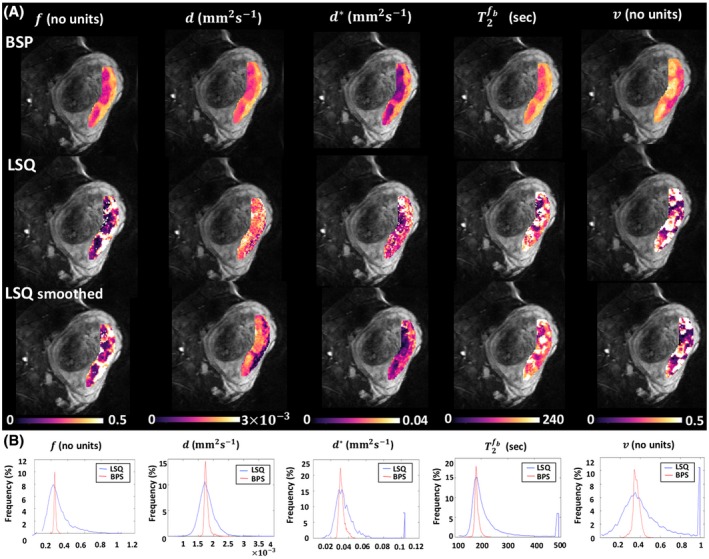
A, DECIDE parameter maps derived from the Bayesian shrinkage prior (BSP) method and least‐squares (LSQ) method with and without data smoothing. B, Histograms of DECIDE voxel estimates for the same data. Curves are histograms for LSQ parameter estimates (blue) and BSP parameter estimates (red)

Figure 6A shows an example of the parameter maps obtained with the BSP and LSQ approaches with and without smoothing. All LSQ parameter maps appeared noisy and artifact‐prone, where BSP fitting notably improved all the parameter maps. $T_{2}^{f_{b}}$ map obtained with LSQ gives a visibly noisy image, while BSP estimate notably improved the resulting $T_{2}^{f_{b}}$ parameter map. $d^{*}$ appeared to be the worst affected and is consistent with observations previously reported in the literature.^24^ The right‐hand maps show the root mean‐squared errors for the three approaches (see the corresponding figure in Supporting Information). Figure 6B shows corresponding histograms. The proportions of the LSQ estimates in the edge‐most bins are 7.5% for $d^{*}$, 5.6% for $T_{2}^{f_{b}}$ and 10.7% for v.

**Figure 7:**
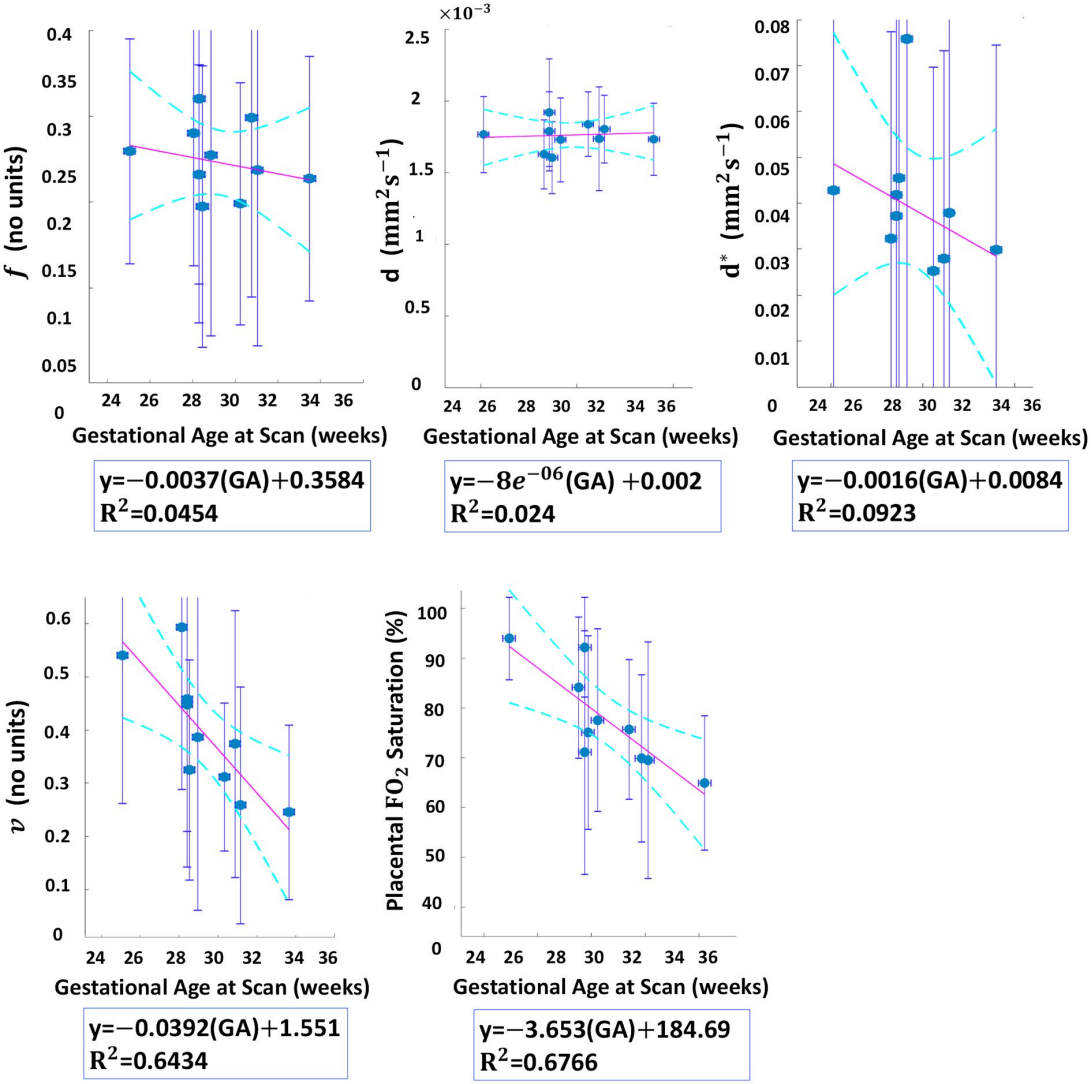
Changes in measured DECIDE parameters with respect to gestational age (GA) after individual LSQ model fitting. The circles indicate the mean values and the error bars represent the *SD*

**Figure 8:**
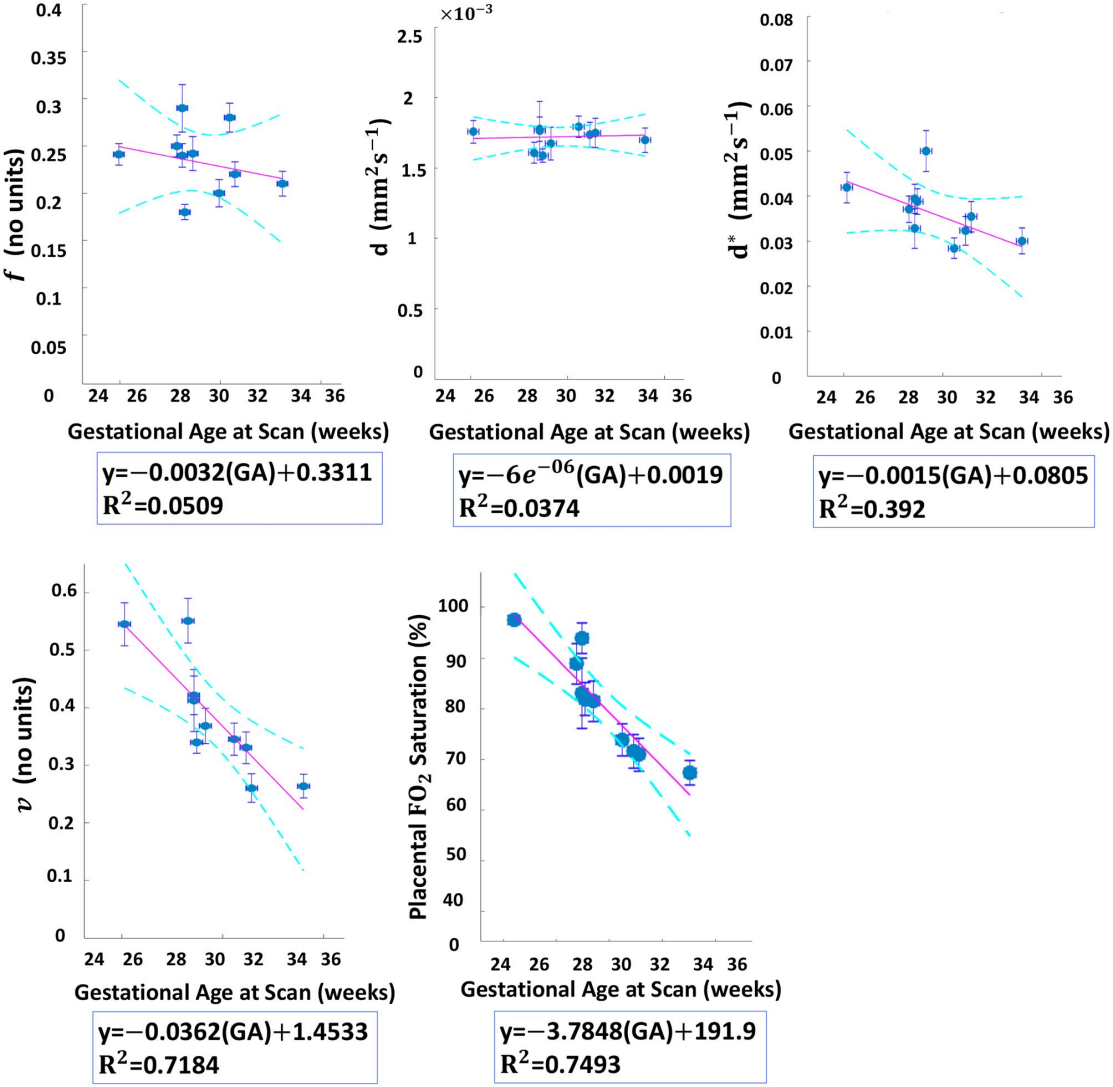
Changes in measured DECIDE parameters with respect to gestational age (GA) after individual BSP model fitting. The circles indicate the mean values and the error bars represent the *SD*

The placenta ROI mean estimated values of the DECIDE parameters with respect to gestational age using the LSQ and BSP methods are presented in Figures 7 and 8, respectively. The mean estimates are broadly consistent between the two approaches. However, the BSP method lead to a decrease of error in all estimated parameters. Significant linear trends are observed for v (*p* = 0.001) and feto‐placental oxygen (FO_2_) saturation measurements (*p* = 0.0004) which both appeared to reduce with increasing gestational age. Measurements of f and d are not observed to change significantly with respect to gestational age.

## 4 DISCUSSION

We have described a framework for motion correction and parametric model fitting applied to quantitative placenta imaging data. We proposed an iterative model‐based registration method for quantitative imaging series. MDR uses pairwise coregistration of source images to the model fit results, avoiding the problem of changes in image contrast between images of the series affecting motion correction. In this study, MDR performance was compared to PPCR.^12^ Important methodological differences between the two methods lie in the fact that they used different approaches to generate target images. The MDR method makes use of the physical signal‐model DECIDE where the PPCR is based on PCA. In order to facilitate a fair comparison among the two registration methods, we kept the FFD control point spacing and transformation model the same. Results from simulated motion show that MDR can compensate for important misalignments due to significant motion corruption. Results from clinical data showed that registration with MDR effectively eliminates motion‐induced blurring, leading to sharp delineation of organ boundaries. Moreover, the MDR method allowed improved motion correction demonstrated by the reduction of the residual bias between the data and the fit. The linear fitting led to a large overall reduction in computation time with a factor of 250. However, the total saving will depend on computer hardware and number of dynamic images in the data. One should note that the linear voxelwise DECIDE fitting was used to generate the series of target imaging volumes for the registration only. The final quantitative parameter estimation was performed nonlinearly by minimizing the sum of squared error between the fitted signal and acquired signal (see Section 2.3.4).

We further described a Bayesian estimation approach for robust estimation of the DECIDE parameters and their summary statistics in the placenta. Results shown here demonstrated that ROI mean estimated values for normal placentas derived using LSQ approach are comparable to the BSP estimates but that the precision of the parameters has been improved. The effect of smoothing the source images has also been examined. Similar to^24^ results demonstrate that while isolated voxels with large errors tend to be removed, non‐isolated voxels remain and may worsen. Smoothing appears to improve the errors on d and $d^{*}$ at the cost of a loss in image resolution. Our method preserves placental parametric heterogeneity but does not implicitly include this in the estimation of the population trends. This enables a more precise estimation of population trends in the data with increasing gestational age or pathology.

The proposed registration method and Bayesian fitting approach are essentially tissue independent and therefore applicable to other organs. A key advantage of the BSP method is that there are no user‐defined parameters, so heterogeneity measures over ROIs are determined by the data alone. Therefore BSP method can be applied to other physical models of quantitative imaging data and help to establish normal changes in quantitative imaging parameters.

Another important finding of this study is the linear correlation of the estimated parameters with the gestational age. Results from the BSP fitting approach suggest that the influence of gestational age on MRI parameters should be taken into account. Our results showed linear correlations between the DECIDE estimated parameters and gestational age, although for a wider range of gestational age, non‐linear models may be more appropriate.^4^ A wider range of gestational age would help demonstrate the longitudinal trend between the MR parameters during pregnancy which might aid in the prediction of obstetric outcomes. Significant negative correlation was found between gestational age and the placental fetal oxygen saturation. This is line with results from a previous invasive study^33^ that examined ranges of blood gas and acid–base measurements over a wide range of gestational age.

It is known that placental saturation values measured by cordocentesis or chorionic sampling decrease with increasing gestational age; however, our model makes several assumptions about placental function that approximate the complexity of the organ which may result in inaccurate values including the high saturation values seen at earlier gestation age. These assumptions include homogenous low‐flow velocity and fixed saturation of the maternal blood pool, and rapid capilliary flow in the fetal blood pool. It is likely that these represent simplifications of the complexity of blood passage within and around the placental villous tissue. We also assumed that maternal blood in the intervillous space has a fixed $T_{2}$ and that saturation does not vary throughout the intervillous space. Since the oxygen extraction by the fetus will always be greater than zero, a concentration gradient is always necessary, depending on the blood delivery conditions and blood velocity through the intervillous space. Deeper in the intervillous space the $T_{2}$ of blood will be somewhere between arterial and venous saturation level as suggested by $T_{2}^{*}$‐weighted images of the placenta.^34^ Assuming that fetal oxygen extraction changes with gestational age, variations in the gradient of maternal blood saturation may influence our estimates of fetal blood saturation. Total blood delivery to the placenta and continued remodeling of the spiral arteries will also cause the interaction of these blood pools to change dynamically with gestation. It is probable that our linear model of changes with gestational age is also inaccurate, but nonetheless it is the most reasonable model to fit with the data that we have. Future studies with larger numbers of subjects or highly sampled longitudinal data will allow models with higher degrees of variation to be fitted and allow us to understand placental maturation in more detail. Further validation work is also needed to investigate the precision of these current assumptions.

The BSP fitting algorithm is independent of the size and shape of the ROI. In general, ROIs between matched placenta of individuals can be considered comparable and parametric distributions can be estimated from the population in the same way as described before for an individual. The interpretation of the multivariate Gaussian in Equation (7) is one formed form the population distributions of parameters rather than those from a single subject ROI. This strategy will produce robust parameter estimates from a matched population and establish a framework for robust longitudinal fitting.

In this study, we have described a comprehensive framework for measuring robust longitudinal trends in MR measurement of placenta perfusion and fetal oxygenation in normal placentas. The framework consists of a new model‐based registration method and a Bayesian estimation approach for jointly estimating voxelwise DECIDE parameters and their summary statistics from DW‐MRI data. This may help us to refine knowledge of changes in MRI properties with increasing gestational age in pregnancies affected by abnormal placentation.

## ACKNOWLEDGEMENTS

We thank our patient and public advisory group for their time and input, and all the families who agreed to take part in this research.

## Supporting information


**FIGURE S1** A, DECIDE parameter maps derived from the Bayesian shrinkage prior (BSP) method and least‐squares (LSQ) method with and without data smoothing. The right‐hand maps show the root mean squared (RMS) errors with the three aproaches. B, Histograms of DECIDE voxel estimates for the same data. Curves are histograms for LSQ parameter estimates (blue) and BSP parameter estimates (red)

## APPENDIX A

The voxelwise parameter values *θ*, and the ROI hyperparameters shared among voxels, μ and $\Sigma_{\mu }$ were updated as follows:

1. Update the mean hyperparameter according to $\mu^{j+1}∼\mu |\Sigma_{\mu }^{j},y$.
2. Update the covariance hyperparameter $\Sigma_{\mu }^{j+1}∼\Sigma_{\mu }|\theta^{j},\;\mu^{j},\;y$.
3. For each voxel, generate a proposal voxelwise parameter, $\theta_{i}^{*}$, by sampling $\theta_{i}\in$ ($f,\;d,\;d^{*},\;r_{2}^{f_{b}},\;v$) in turn from $N(\theta_{i},\;w_{i}\theta_{i}^{2})$, where $w_{i}$ is a proposal variance for parameter i.
4. Update $\theta_{i}^{j+1}$ with this new value if $r∼U\left(0,\;1\right)\;<\;\alpha \left(\theta_{i}^{*},\;\theta_{i}^{i}\right)$, otherwise $\theta_{i}^{j+1}\;=\;\theta_{i}^{j+1}$.

Proposal variances, $w_{i}$, are initialized at $w_{i}\;=\;0.5$ for all i. Periodically these are tuned to ensure approximately 25% of samples are being accepted per.^32^ The acceptance probability, α, is given by $\alpha \left(\theta^{*},\;\theta^{0}\right)\;=\;\frac{p(y|\mu ,\;\Sigma_{\mu },\;\theta \;=\;\theta^{*})}{p(y|\mu ,\;\Sigma_{\mu },\;\theta \;=\;\theta^{0})}$. That is, α is higher when the proposal $\theta^{*}$ better matches the observed data than $\theta^{0}$.
