# Supplementary figures and images for: Improved fetal blood oxygenation and placental estimated measurements of diffusion‐weighted MRI using data‐driven Bayesian modeling
Source: Magn Reson Med. 2019 Nov 19;83(6):2160–72. doi: 10.1002/mrm.28075 (PMC7064949; doi:10.1002/mrm.28075)

**(a)**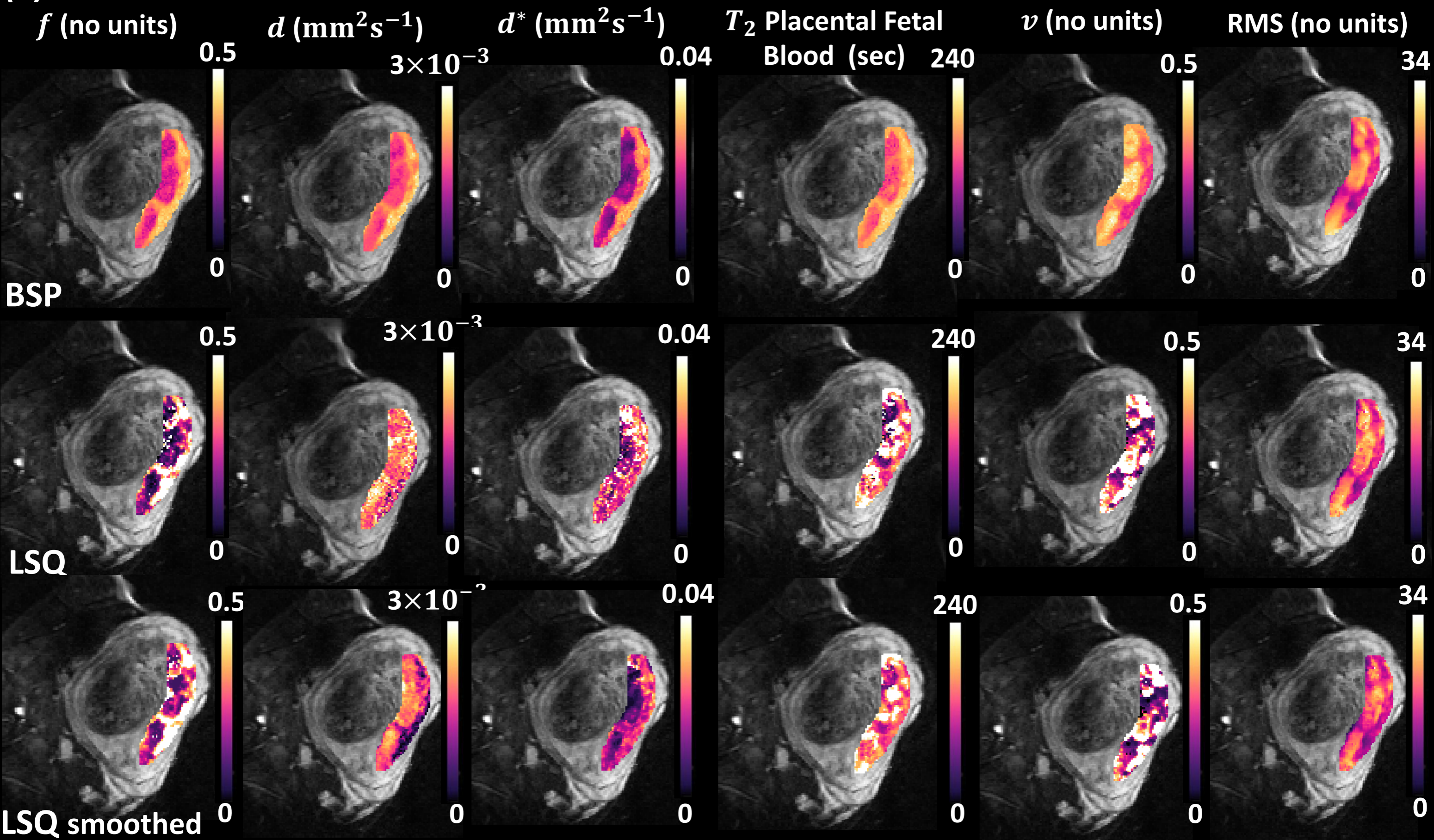**(b)**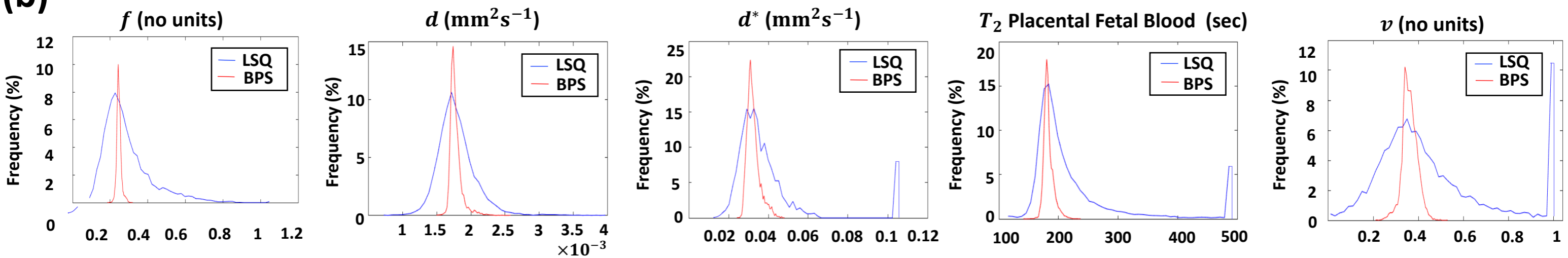

Supplement: Supplementary file 1 — FIGURE S1 A, DECIDE parameter maps derived from the Bayesian shrinkage prior (BSP) method and least‐squares (LSQ) method with and without data smoothing. The right‐hand maps show the root mean squared (RMS) errors with the three aproaches. B, Histograms of DECIDE voxel estimates for the same data. Curves are histograms for LSQ parameter estimates (blue) and BSP parameter estimates (red) [file MRM-83-2160-s001.pdf]
